# Oxytocin may be useful to increase trust in others and decrease disruptive behaviours in patients with Prader-Willi syndrome: a randomised placebo-controlled trial in 24 patients

**DOI:** 10.1186/1750-1172-6-47

**Published:** 2011-06-24

**Authors:** Maïthe Tauber, Carine Mantoulan, Pierre Copet, Joseba Jauregui, Genevieve Demeer, Gwenaëlle Diene, Bernadette Rogé, Virginie Laurier, Virginie Ehlinger, Catherine Arnaud, Catherine Molinas, Denise Thuilleaux

**Affiliations:** 1 Centre de Référence du Syndrome de Prader-Willi, Division of Endocrinology, Genetics, Gynaecology and Bone Diseases, Hôpital des Enfants, Toulouse, France; 2 INSERM; Centre de Physiopathologie de Toulouse-Purpan, UMR 1043; F-31059 Toulouse, France; 3 Université de Toulouse; UPS; Centre de Physiopathologie de Toulouse-Purpan, Place du Dr Baylac, F-31059 Toulouse, Cedex 9, France; 4 Hôpital Marin d'Hendaye, Hendaye, France; 5 Unité de Recherche Interdisciplinaire Octogone EA4156, CERPP, Toulouse, France; 6 Epidémiologie, Equipe d'Epidémiologie Clinique, CHU Toulouse, UMR INSERM U1027, Université Paul Sabatier, Toulouse, France

## Abstract

**Background:**

Prader-Willi syndrome (PWS) is a complex neurodevelopmental genetic disorder with hypothalamic dysfunction, early morbid obesity with hyperphagia, and specific psychiatric phenotypes including cognitive and behavioural problems, particularly disruptive behaviours and frequent temper outbursts that preclude socialization. A deficit in oxytocin (OT)-producing neurons of the hypothalamic paraventricular nucleus has been reported in these patients.

**Methods:**

In a double-blind, randomised, placebo-controlled study, 24 adult patients with PWS received a single intranasal administration of 24 IU of OT or placebo and were tested 45 min later on social skills. Behaviours were carefully monitored and scored using an in-house grid as follows: over the two days before drug administration, on the half-day following administration, and over the subsequent two days. All patients were in a dedicated PWS centre with more than ten years of experience. Patients are regularly admitted to this controlled environment.

**Results:**

Patients with PWS who received a single intranasal administration of OT displayed significantly increased trust in others (P = 0.02) and decreased sadness tendencies (P = 0.02) with less disruptive behaviour (P = 0.03) in the two days following administration than did patients who received placebo. In the half-day following administration, we observed a trend towards less conflict with others (p = 0.07) in the OT group compared with the placebo group. Scores in tests assessing social skills were not significantly different between the two groups.

**Conclusions:**

This study needs to be reproduced and adapted. It nevertheless opens new perspectives for patients with PWS and perhaps other syndromes with behavioural disturbances and obesity.

**Trial registration number:**

ClinicalTrials.gov: NCT01038570

## Introduction

Prader-Willi syndrome (PWS) is a rare, complex neurodevelopment genetic disorder arising from the lack of expression of paternally inherited imprinted genes on chromosome 15q11-q13 [1]. The syndrome includes severe neonatal hypotonia with suckling difficulties, dysmorphia, early onset of hyperphagia and morbid obesity, endocrine dysfunctions, learning disabilities, and behavioural, social and psychiatric disturbances, leading to severe consequences and difficult management issues [2].

Patients with PWS display compulsive and ritualistic behaviour [3], pronounced emotional lability, and a striking inability to control emotion, which results in frequent temper outbursts and precludes normal socialisation [4]. The anger often seems to be an expression of frustration and the feeling of not being understood, but it may also be due to an impaired capacity to understand the motivations of others in the social milieu [5]. These patients display poor social adjustment, with poor peer relationships, withdrawal tendencies, and some features of the autism spectrum.

Interestingly, a deficit in the oxytocin (OT)-producing neurons of the paraventricular nucleus in the brain of these patients was reported [6]. OT was identified as a key neuropeptide involved in social interactions by enhancing peer recognition and bonding behaviour in numerous species [7]. In humans, nasal OT administration improves emotion recognition and face processing in healthy and autistic individuals. OT appears to be the main neurohormone to explain empathy and theory of mind (TOM) [8-14]. TOM is a concept describing the cognitive attribute that allows us to understand the thinking of others and to take into account their views [15], while empathy is part of the interpersonal processes that are crucial for healthy social and moral development [16]. We therefore hypothesized that this deficit in the oxytocin (OT)-producing neurons may be related to OT dysfunction, which would explain at least in part the inability of these patients to control emotions and their poor social adjustment, which in turn might explain their unpredictable disruptive behaviours and frequent temper outbursts.

Given the possible OT deficit and the overlap between PWS and autism reported elsewhere [5], it seemed logical to evaluate the effect of OT administration in these patients. We hypothesised that OT would improve the behaviour of patients with PWS by improving their ability to read social cues, thereby facilitating their socialisation.

## Materials and methods

We designed a double-blind randomised, placebo-controlled pilot study (ClinicalTrials.gov Identifier: NCT01038570) to be conducted in a dedicated PWS centre, where patients are regularly admitted for one month and live in a controlled environment. Patients know they have no free access to food but no doubt about the time and the content of meals. For each stay, 16 patients with PWS are admitted and take part in daily planned occupational and physical group activities. They also receive medical care as needed and psychological support. The 24 patients included in this study (16 females, 8 males, median age 28.5 years [18.7 to 43.6], median BMI 43 kg/m^2 ^[19.4 to 67.4]) were stratified on gender and IQ (median IQ = 51[45 to 75]). The diagnosis of PWS was genetically confirmed using the standard DNA methylation test and subsequent molecular analyses showed a classic genotype distribution. Nineteen patients had a deletion (79%), three (12.5%) a UPD, and in two cases (8.5%) the genetic subtype was unknown. Patients as well as parents or caregivers gave their written consent prior to entering the study. Exclusion criteria were an abnormal ECG and other severe cardiovascular problems. Patients were well known to the team. Each pair of patients of the same gender and same IQ range was evaluated the same day. The two patients of the pair received either OT or placebo in a double-blind randomisation. Patients were included in the study over the course of three series of stays.

Each patient received a single intranasal administration of either placebo (saline solution) or OT (Syntocinon^®^/- Spray, Novartis, Basel, Switzerland) with three puffs per nostril (24 UI). The dose of 24 UI is the most frequently reported in the literature. As we did not succeed in obtaining empty flasks of Syntocinon^® ^from Novartis, we used a different flask and the drug was administered by a nurse from another department of the hospital. This nurse knew neither the patients nor the study.

The behaviour of the patients was carefully monitored, scored daily by the team psychologist, and documented on the case report form for the two days before drug administration, on the half-day following administration (early effects) and over the two days following drug administration (late effects). Both the staff and the rater were unaware of treatment status. There are currently no validated grids that evaluate the specific behavioural features of patients with PWS that we particularly wanted to study, *i.e*. tendencies towards isolation, sadness, and depression, self-depreciation, self-mutilation, conflicts with others, disruptive behaviour, interest in friendship, interest in love affairs, and trust of others. For this reason, we used an in-house grid developed by the caregivers and based on the routine observation of these patients in this dedicated centre for PWS with recognized expertise [17,18]. The criteria that the staff used to fill in the grid are explained in Table 1. The patients were well known to the team but the mean behavior status was re-evaluated during the first week of admission using the same grid. The behavioural features of the patients were scored as unchanged, moderately changed, or severely changed. For each criterion, a negative score reflected deterioration in the mean behavior status of the patient while a positive score reflected an improvement. Eating behaviour was also scored using three categories: usual, better than usual or worse, based on the analysis of each meal. The following were evaluated: amount of food intake per meal, amount of food requested per meal, duration of meal, and behaviour before/during/after the meal. To evaluate the early effect of OT, we analysed only the first meal after drug administration.

**Table 1:** Criteria used by the staff to fill out the behavioural grid

Behavioural items	Scoring criteria
Isolation tendencies	playing solitary games, taking walks alone
Sadness tendencies	mood state expressed by tears, complaints, frustration, irritation with others
Depressive tendencies	remaining in bed, neglecting self-care, showing little motivation or interest, withdrawn
Self-depreciation	belittling self, pointing out own incapacities and failings, expressing low self-value
Self-mutilation	mutilations: scratching or scraping off skin, pulling out hair or eyebrows
Conflicts with others	opposing others, verbal or physical disputes, other-directed complaints, making threats
Disruptive behaviour	temper tantrums, sulking, running away, slamming doors, isolating self in room, breaking things, etc., in response to a conflict or a frustration
Interest in friendship	making friends with other patients, taking part in group activities
Interest in love affairs	showing interest in having a special relationship with someone of the opposite sex (or not...)
Trust in others	participating in group activities, talking with others while taking walks, spontaneously greeting others, introducing self to caregivers or asking for help, etc.

Forty-five minutes after the intranasal administration, three tests evaluating the understanding of social codes were administered, which lasted one hour. Conversely to the behavioural grid, there was no pre-administration evaluation. The Sally and Ann test assesses one's ability to understand simple situations by story-telling and pictures [19]. Cartoons depicting more complex social situations designed for the evaluation of autistic children were also used ("Cartoons", unpublished test). The "Reading the Mind in the Eyes" test (RMET) assesses the ability to read emotions from subtle affective facial expressions, especially in the eyes. A shortened test with 19 images from the 36-item revised version [20] was chosen by the team psychologist. In these three tests, TOM is necessary to understand and analyse the social situations that are presented. Statistical analysis was performed using the Mann-Whiney test, the Wilcoxon test or the chi2 test.

Four patients in the OT group and three in the placebo group were receiving psychotropic medications.

## Results

Patients in the OT group displayed significantly increased trust in others (P = 0.02), fewer tendencies towards sadness (P = 0.02) and less disruptive behaviour (P = 0.03) in the two days following intranasal drug administration. They also showed a tendency towards fewer conflicts with peers (P = 0.07) on the half-day following intranasal administration. Table 2 shows these results in detail. Of note, there was no difference between the two groups before intranasal administration for any item.

**Table 2:** Behavioural scores pre-and post-administration of OT in the placebo and OT groups; pre-administration score is the mean of the scores reported on the two days before administration; Early effect is the immediate score recorded the half-day following administration and late effect is the mean of the scores reported on the two days following administration (the immediate score recorded the half-day following administration was excluded).

Variable	Placebo Group	OT Group	*P-value*
**Isolation tendencies**			
Pre-administration	-0.111 ± 0.296	-0.083 ± 0.289	0.580
Early effect	-0.250 ± 0.452	-0.083 ± 0.289	0.284
Late effect	-0.111 ± 0.217	0 ± 0	0.070
**Sadness tendencies**			
Pre-administration	-0.208 ± 0.276	-0.194 ± 0.407	0.513
Early effect	-0.083 ± 0.289	-0.083 ± 0.289	>0.99
Late effect	-0.347 ± 0.379	-0.083 ± 0.289	**0.021**
**Depressive tendencies**			
Pre-administration	-0.028 ± 0.096	-0.056 ± 0.192	0.952
Early effect	0 ± 0	0 ± 0	>0.99
Late effect	-0.125 ± 0.311	0 ± 0	0.149
**Self-depreciation**			
Pre-administration	-0.028 ± 0.096	-0.056 ± 0.192	0.952
Early effect	0 ± 0	-0.083 ± 0.289	0.317
Late effect	-0.083 ± 0.289	0 ± 0	0.317
**Self-mutilation**			
Pre-administration	-0.069 ± 0.166	-0.278 ± 0.468	0.271
Early effect	-0.083 ± 0.289	-0.083 ± 0.289	>0.99
Late effect	-0.028 ± 0.096	-0.083 ± 0.195	0.482
**Conflicts with others**			
Pre-administration	-0.208 ± 0.276	-0.319 ± 0.359	0.467
Early effect	-0.250 ± 0.452	0 ± 0	0.07
Late effect	-0.125 ± 0.237	-0.111 ± 0.205	0.939
**Disruptive behaviour**			
Pre-administration	-0.361 ± 0.354	-0.431 ± 0.344	0.744
Early effect	-0.250 ± 0.452	-0.167 ± 0.389	0.623
Late effect	-0.306 ± 0.382	-0.042 ± 0.144	**0.031**
**Interest in friendship**			
Pre-administration	0.806 ± 0.324	0.875 ± 0.433	0.349
Early effect	0.833 ± 0.389	0.917 ± 0.515	0.683
Late effect	0.778 ± 0.410	1.028 ± 0.234	0.088
**Interest in love affairs**			
Pre-administration	0.847 ± 0.579	0.972 ± 0.531	0.589
Early effect	0.833 ± 0.389	0.917 ± 0.515	0.683
Late effect	0.847 ± 0.441	0.903 ± 0.411	0.714
**Trust in others**			
Pre-administration	0.833 ± 0.389	0.861 ± 0.332	0.929
Early effect	0.917 ± 0.289	0.833 ± 0.389	0.546
Late effect	0.764 ± 0.366	1.028 ± 0.096	**0.023**

In the group of the 19 patients with deletions (9 in the OT group, 10 in the placebo group), a significant effect of intranasal OT on disruptive behaviour was also found (P = 0.04), as well as a trend towards more trust in others (P = 0.05) fewer sadness tendencies (P = 0.07) and more interest in friendship (P = 0.05) (data not shown). The low number of patients with no deletion (n = 5) did not allow statistical analysis (data not shown).

The same analysis was performed excluding the seven patients who received psychotropic medications (4 patients in the OT group and 3 in the placebo group) and showed that the OT group had less disruptive behaviour (P = 0.04) in the two days following intranasal drug administration (data not shown).

The pre-post difference, which was calculated as "post-administration late effect score" minus "pre-administration score", was significant in the OT group for the disruptive behaviour (P = 0.01) and self-mutilation (P = 0.047) items, whereas there was no difference in the placebo group (Table 3). There was a trend (P = 0.07) towards a different evolution in the pre-post difference between the two groups for the disruptive behaviour item.

**Table 3:** Pre-post differences in the behavioural scores of the two groups and change comparison between OT treatment and placebo.

	Placebo group			OT group			OT vs. Placebo
	
Change in behavioural scores	Mean	SD	P-value (Mann Whitney)	Mean	SD	P-value (Mann Whitney)	P-value (Wilcoxon*)
Isolation tendencies	-0.000	0.402	0.723	0.083	0.289	0.317	0.404
Sadness tendencies	-0.139	0.324	0.260	0.111	0.533	0.343	0.217
Depressive tendencies	-0.097	0.230	0.158	0.056	0.192	0.317	0.088
Self-depreciation	-0.056	0.192	0.317	0.056	0.192	0.317	0.166
Self-mutilation	0.042	0.203	0.530	0.194	0.354	**0.047**	0.236
Conflicts with others	0.083	0.314	0.735	0.208	0.498	0.150	0.532
Disruptive behaviour	0.056	0.457	0.812	0.389	0.385	**0.011**	0.070
Interest in friendship	-0.028	0.497	0.690	0.153	0.379	0.183	0.245
Interest in love affair	-0.000	0.632	1.000	-0.069	0.429	0.600	0.704
Trust in others	-0.069	0.399	0.850	0.167	0.333	0.084	0.222

* Wilcoxon test is based on individual changes.

No statistical difference was observed in the scores assessing eating behaviour between the two groups. Nevertheless, five patients in the OT group (45%) and one in the placebo group (10%) said that they did not feel hungry and slightly decreased their food intake over the two days following the intranasal administration.

**Figure 1:**
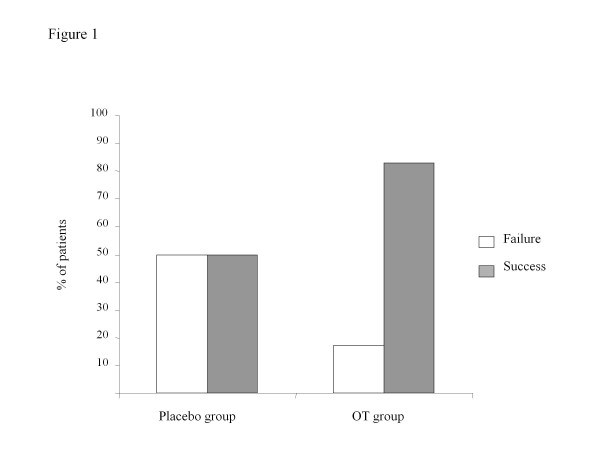
**Percentage of patients in the placebo group in the OT group who failed (white bar) or passed (grey bar) the Sally and Ann test**.

The tests evaluating social skills showed a tendency towards improvement in the OT group (n = 12). Eighty-three percent of the patients in this group successfully completed the Sally and Ann test compared with 50% in the placebo group (n = 12) (P = 0.19, Figure 1). The same tendency was observed with the Cartoons, with a higher score obtained in the OT group than in the placebo group: 3.75 (1-18) *vs*. 6 (0-12) (P = 0.56). There was no significant difference in the total RMET score between the two groups: 7 (4-9) in the OT group vs. 5.5 (1-11) (P = 0.18).

Tolerance of OT was excellent with no effect on electrocardiogram and PSA. No adverse event was observed during the study.

## Discussion

We were able to show for the first time that patients with PWS who received a single intranasal administration of OT showed significantly higher trust in others, fewer sadness tendencies and less disruptive behaviour, which may in turn improve socialization. Patients also showed a tendency towards less conflict with others on the half-day following OT administration. Only late effects were significantly different between the two groups. Moreover, the changes in the disruptive behaviour and self-mutilation scores were significant, whereas no change was observed in the placebo group. Comparisons of the two groups revealed no significant differences in the changes but some trends could be observed (the disruptive behaviour and depression items). The lack of significant difference may have been due to the well-known high inter-individual variability of patients with PWS or the low number of patients.

The lack of statistically significant difference in the behaviour scores on the half-day following OT administration suggests that early effects were not major in these patients, while late effects (over the 2 days following administration) were significant and relevant to both the known actions of OT and the social dysfunctions observed in these patients.

The genetic subtypes of PWS had been shown to partly explain the well-known wide variability in the clinical features. We recently showed that patients with deletions had specific and significant relative weaknesses in the comprehension and picture completion subtests and relative strength in object assembly compared with patients without deletion [17]. This may also explain the results of the tests we used, as 19 out of the 24 patients had deletions. Interestingly, the effect of intranasal OT on disruptive behaviour remained significant in the group of the 19 patients with deletions. The low number of patients (n = 5) without deletion did not allow statistical analysis and further studies are needed with larger series.

The lack of statistically significant difference in the various tests measuring social skills may have been due to the fact that they were performed too early.

We are aware of the weaknesses in the protocol for this pilot trial. For example, we used only one dose without conducting a preliminary dose-finding study. We did not use validated questionnaires for evaluating behaviour because most of the validated questionnaires are difficult to use for individuals with PWS. We thus chose to use our in-house grid. We did not measure plasmatic OT levels mainly because of the known difficulties of sampling in these patients. We recently published a report on the brain imaging abnormalities in patients with PWS, particularly relatively hypoperfused brain areas [21], and it would be interesting to take these findings into account in future OT studies.

In conclusion, this preliminary study suggests that OT may increase trust in others and decrease sadness tendencies and disruptive behaviour in patients with PWS. It is therefore tempting to suggest that OT may be used as a therapeutic option in PWS. In any case, OT, like other hormones, may become a substitutive treatment in PWS. Further dose-effect and long-term studies are needed in larger series combined with functional brain imaging studies.

## Competing interests

The authors declare that they have no competing interests.

## Authors' contributions

MT conceived the study, participated to the design of the study, analyzed the results and wrote the manuscript. CMa participated to the design of the study and performed the study. PC performed the study. JJ participated to the design of the study. GDe performed the study. GDi and BR participated to the design of the study. VL performed the study. VE and CA performed the statistical analysis. CMo participated to the design of the study, performed the study, analyzed the results and wrote the manuscript. DT conceived the study, participated to the design of the study, performed the study, analyzed the results and wrote the manuscript. All authors read and approved the final manuscript.
